# The surgical management of a hypogastric dermatofibrosarcoma protuberans

**DOI:** 10.11604/pamj.2019.34.116.17740

**Published:** 2019-10-29

**Authors:** Leila Essid, Selim Sassi, Khaled Khelil, Mohamed Ali Sbai, Riadh Maalla

**Affiliations:** 1 Orthopaedic and Trauma Department, Maamouri Hospital, Nabeul, Tunisia; 2 General Surgery Department, Maamouri Hospital, Nabeul, Tunisia; 3 Orthopaedic and Trauma Department, Rabta Hospital, Tunis, Tunisia

**Keywords:** Dermatofibrosarcoma, skin defect, plastic surgery, flap

## Abstract

Dermatofibrosarcoma protuberans (DFSP) is an uncommon skin tumor with intermediate grade of malignancy. Wide surgical resection with disease-free margin offers an excellent probability of cure. These large excisions cause large skin defect that can be difficult to cover. Through this case report we describe a surgically treated DFSP that was covered by a Mc Gregor flap; which despite its seniority is still relevant and continues to serve plastic surgery.

## Introduction

Dermatofibrosarcoma protuberans (DFSP) is a rare skin tumor. This tumor belongs to the groups of fibroblastic or myofibroblastic tumors with intermediate malignancy according to the latest classification of WHO in 2013 [1]. Its particularity and gravity are linked to its local aggressivity and its high rate of recurrence. DFSP often affects the young adult and has a predilection for the trunk followed by the proximal ends and then the head and neck [2]. Surgery have the major role in the curative treatment of DFSP, and its prognosis depends on the quality of the initial excision. These large excisions cause a large skin defect sometimes difficult to cover and requires the use of free flaps. We present a case of surgically treated DFSP in which the skin defect was covered by a Mc Gregor flap which despite its seniority is still relevant and continues to serve plastic surgery.

## Patient and observation

**Figure 1:**
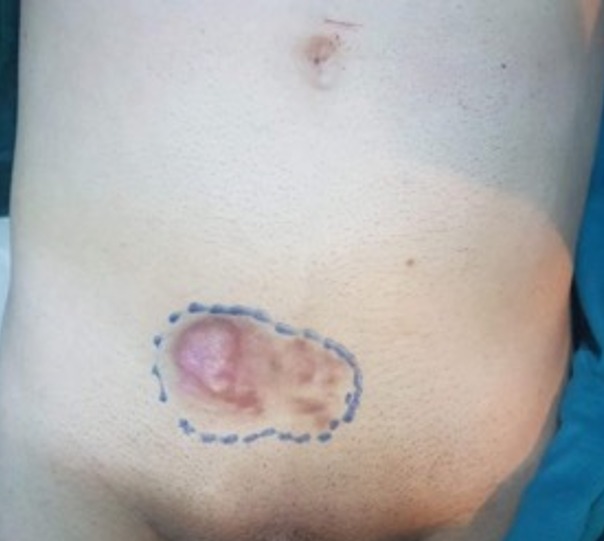
Dermatofibrosarcoma protuberans (DFSP) presenting as an induration with multiple nodules

**Figure 2:**
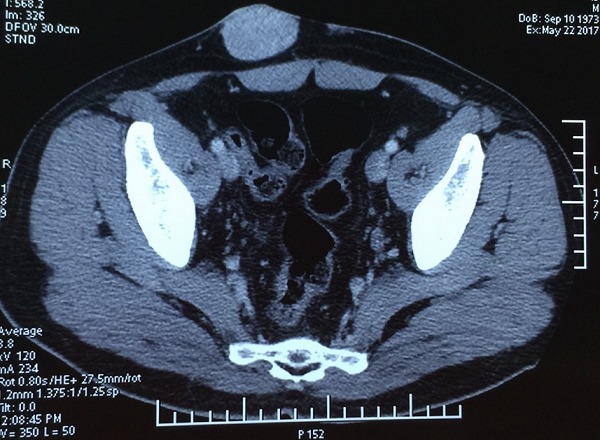
CT showing the lesion

**Figure 3:**
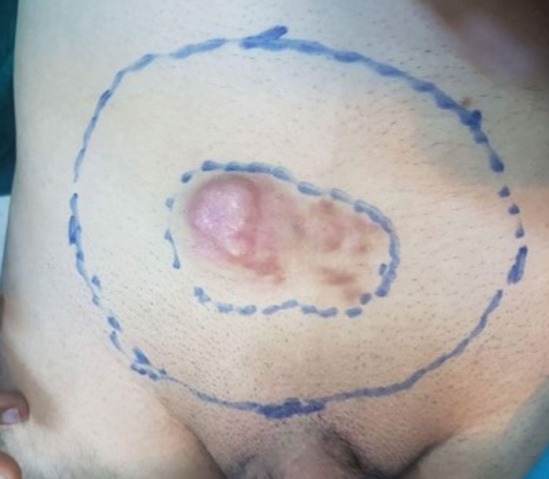
Margin of the excision of 5 cm

**Figure 4:**
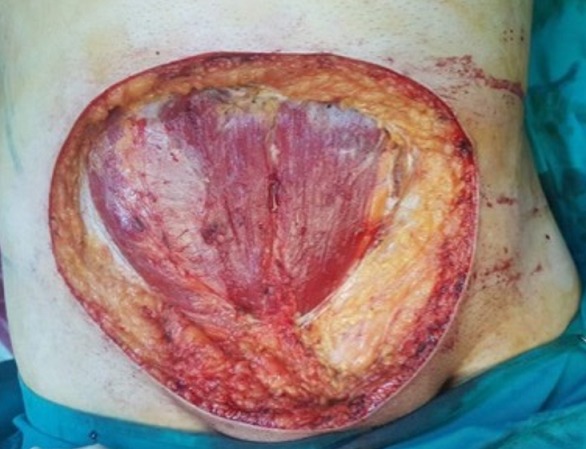
Large skin defect post excision

**Figure 5:**
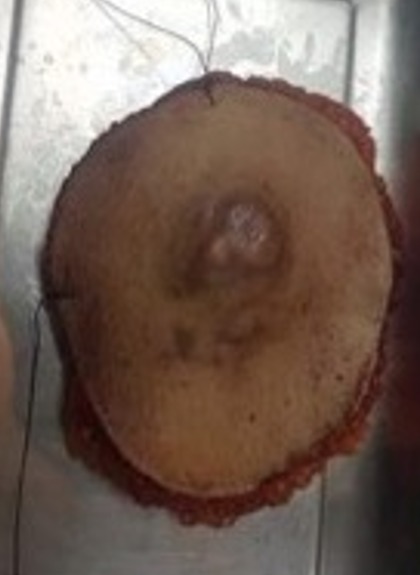
The macroscopic aspect of the excision

**Figure 6:**
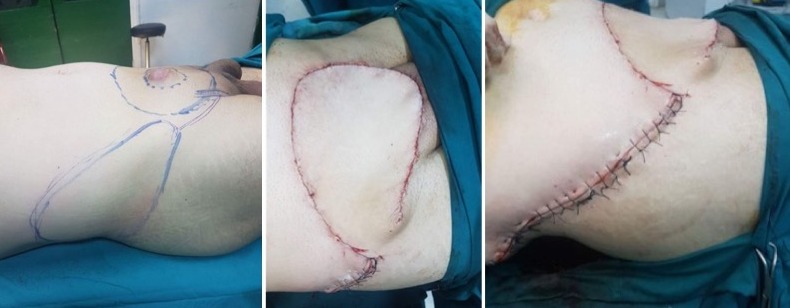
McGregor flap used to cover the skin defect after excision

**Figure 7:**
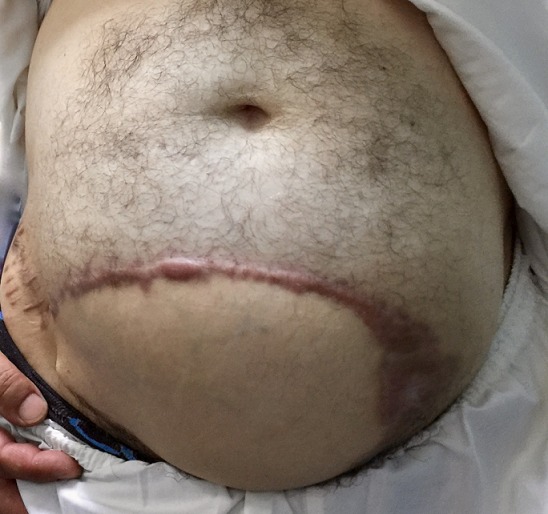
The result 1 year and a half after surgery

A 45-year-old man with a hypogastric tumor evolving for 5 years, neglected by the patient. It was a small nodule that gradually increased in volume without any pain or functional inconvenience (Figure 1). The biopsy of the lesion concluded to a DFSP. Computed tomography (CT) showed infiltration of the subcutaneous fat without extension to the underlying muscles (Figure 2). The excision was made with lateral margins of 5 cm and carrying deeply the fascia of the internal oblique and the right rectus muscle (Figure 3, Figure 4, Figure 5). The cutaneous defect was covered at the same time by an inguinal Mc Gregor flap and the donor zone was sutured directly (Figure 6). The histopathological exam concluded to a DFSP with a healthy limit of excision. The clinical aspect at 1.5 year follow-up showing the absence of recurrence with an excellent aesthetic result (Figure 7).

## Discussion

Surgery plays a major role in the curative treatment of DFSP. The main challenge facing this tumor, which projects asymmetric and subclinical extensions, is to obtain healthy surgical margins [3]. The two techniques discussed are wide excision and micrographic surgery [4]. The micrographic surgery makes it possible to study the entire tumor circumference with reduction of the margins of excision but remains not practiced in most countries. In locally advanced, inoperable or metastatic forms, treatment is based on targeted therapies such as imatinib mesylate (Glivec^®^), which is currently approved for use in this indication. Post-excision reconstruction involves all the therapeutic arsenal of plastic and reconstructive surgery. The choice will depend on the site and the size of the defect as well as the exposure or not of the underlying noble elements. The thin skin graft is indicated in case of loss of substance without exhibition of noble elements. The use of free flaps, in particular the Latissimus Dorsi Flap, which is increasingly practiced because of its reliability and its large size, makes it possible to cover large defects [5]. In our case we have opted for a Mc Gregor flap [6], a flap described since 1972, often forgotten but still reproducible and reliable. It allows to have a large skin palette without sacrificing an important vascular axis with the possibility of a direct closure of the donor area, leaving a hidden scar.

## Conclusion

Wide and deep surgical excision with healthy margins remains the cornerstone of DFSP treatment. The micrographic technique is certainly less mutilating but remains not practiced in our country. Despite its age and the advent of several microsurgical techniques, the Mc Gregor flap is still valid and resolves large skin defect problems with a simple, reliable and one-time surgery.

## Competing interests

The authors declare no competing interests.
